# Identification of a lead like inhibitor of the hepatitis C virus non-structural NS2 autoprotease

**DOI:** 10.1016/j.antiviral.2015.10.001

**Published:** 2015-12

**Authors:** Joseph Shaw, Mark Harris, Colin W.G. Fishwick

**Affiliations:** a School of Chemistry, University of Leeds, Leeds, LS2 9JT, United Kingdom; b School of Molecular and Cellular Biology, Faculty of Biological Sciences, University of Leeds, Leeds, LS2 9JT, United Kingdom; c Astbury Centre for Structural Molecular Biology, University of Leeds, Leeds, LS2 9JT, United Kingdom

**Keywords:** Hepatitis C virus, NS2, Small molecule inhibitor, Autoprotease, HCV, Hepatitis C virus, NS, non-structural, SGR, subgenomic replicon, DAA, direct acting antiviral, JFH1, Japanese Fulminant Hepatitis 1, EC_50_, 50% effective concentration, LarI, luciferase assay reagent 1, PLB, Passive Lysis Buffer

## Abstract

Hepatitis C virus (HCV) non-structural protein 2 (NS2) encodes an autoprotease activity that is essential for virus replication and thus represents an attractive anti-viral target. Recently, we demonstrated that a series of epoxide-based compounds, previously identified as potent inhibitors of the clotting factor, FXIII, also inhibited NS2-mediated proteolysis *in vitro* and possessed anti-viral activity in cell culture models. This suggested that a selective small molecule inhibitor of the NS2 autoprotease represents a viable prospect. In this independent study, we applied a structure-guided virtual high-throughput screening approach in order to identify a lead-like small molecule inhibitor of the NS2 autoprotease. This screen identified a molecule that was able to inhibit both NS2-mediated proteolysis *in vitro* and NS2-dependent genome replication in a cell-based assay. A subsequent preliminary structure–activity relationship (SAR) analysis shed light on the nature of the active pharmacophore in this compound and may inform further development into a more potent inhibitor of NS2 mediated proteolysis.

## Highlights

• In silico screening identified a small molecule inhibitor of hepatitis C virus NS2 autoprotease *in vitro*.
• This compound showed specific inhibition of NS2-NS3 autocleavage dependent HCV genome replication in a cell based assay.
• NS2 protease is a valid target for further drug development.

## 1 Introduction

Hepatitis C virus (HCV) infects 2.8% of the worldwide population, with 85% of cases progressing to chronic infection (Webster et al., 2015), associated with both liver cirrhosis and hepatocellular carcinoma. HCV possesses a 9.6 kb positive sense RNA genome which encodes a single 3000 amino acid polyprotein and viral replication is absolutely dependent on proteolytic processing of this polyprotein into mature proteins. Host proteases cleave between the virion structural proteins (Core, E1 and E2), the p7 viroporin and p7-NS2 junction. Two virally-encoded protease activities complete processing of the remaining non-structural (NS) proteins NS2, NS3, NS4A, NS4B, NS5A and NS5B. An autoprotease activity performs cleavage at the NS2-NS3 junction, while the remaining junctions are processed by the NS3 serine protease.

HCV therapy has advanced rapidly with the development of direct acting anti-virals (DAAs) which target polyprotein processing or genome replication. DAAs target two virally encoded enzymes; the NS3 protease and the NS5B RNA-dependent RNA polymerase, but also NS5A (which lacks enzyme activity) through a poorly defined mode of action (Webster et al., 2015). While the success of these therapies has been encouraging to date, the long term response and potential for viral resistance remain to be seen.

Until recently, the only HCV encoded enzyme activity to which small molecule inhibitors have not been reported is the autoprotease activity encoded within NS2 and responsible for cleavage at the NS2-NS3 junction. NS2 is a 23 kDa protein that in addition to autoprotease activity has an independent, albeit poorly defined, role in virus assembly (Jones et al., 2007, Ma et al., 2011, Shanmugam et al., 2015). It comprises an N-terminal transmembrane domain and a C-terminal cytoplasmic domain, the latter of which contains the cysteine autoprotease activity. The enzyme activity is encoded within NS2 but is enhanced by elements from the NS3 N-terminus (Schregel et al., 2009). NS2-NS3 cleavage is essential in the virus lifecycle (Jones et al., 2007, Kolykhalov et al., 2000, Welbourn et al., 2005). Although NS2 plays no direct role in genome replication processing of NS3 from an NS2-NS3 precursor is required for an NS3-mediated function which is essential in the establishment of replication complexes (Isken et al., 2015).

Due to its essential nature in the HCV lifecycle, the NS2 autoprotease represents an attractive therapeutic target (Lorenz, 2010, Rice, 2011). In this regard, we recently reported that a series of epoxide-containing small molecule inhibitors of the transglutaminase FXIII-A (Avery et al., 2015) were also able to inhibit NS2 mediated proteolysis both *in vitro* and in cell-based systems (Shaw et al., 2015). To further explore the potential to inhibit the HCV NS2 autoprotease a structure-guided virtual high-throughput screening approach was employed to identify a lead-like small molecule inhibitor. This molecule represents a first-in-class anti-viral agent with activity against infectious HCV in cell culture and provides evidence that inhibitors of virally encoded auto-proteases are a viable prospect.

## 2 Materials and methods

### 2.1 Compounds

Compounds **160** (ID: 38490315), **160–3**, **160–4**, **160–5**, **160–6**, **160–7, 160–8** and **160–9** were from ChemBridge Corporation. SM-1, SM-2 and Telaprevir were from KeyOrganics, Sigma Aldrich and MedChem Express respectively. **160–1** and **160–2** were synthesised in-house (Supplementary Materials). All compounds were confirmed using a VG Autospec mass spectrometer with electron spray ionisation (ES) at 70 eV.

### 2.2 *In silico* enrichment of screening libraries

C-terminal residues were sequentially removed from the active site cavity of the post-cleaved NS2 protease domain structure (PDB: 2HD0) using Maestro (Schrodinger) to produce the models NS2Δ_P1−P2_, NS2Δ_P1−P5_ and NS2Δ_P1−P10_. Virtual screening was performed using eHITS (SymBioSys) to dock and score a library of >5 × 10^5^ commercially available lead-like molecules. Additionally, SPROUT (Gillet et al., 1994) was employed to construct models of molecules predicted to form favourable interactions. Top scoring molecules were expanded using ROCS 3.2.0.4 (OpenEye Scientific Software, Santa Fe, NM. http://www.eyesopen.com) (Hawkins et al., 2007) and filtered by favourable modelled binding pose, structural diversity and cLogP to yield a library of 200 compounds.

### 2.3 Screening of small molecules *in vitro*

NS2-NS3-FLAG (JFH-1 isolate, genotype 2a) was purified, and proteolysis reactions were performed as previously described (Shaw et al., 2015). Compounds were added to reactions prior to addition of NS2-NS3-FLAG by dilution from DMSO stocks (final DMSO 0.75%). Reactions were incubated at room temperature for 16 h, terminated by addition of Laemmli buffer and the NS3-FLAG proteolysis product quantified by western blot using M2 anti-FLAG monoclonal antibody (Sigma Aldrich) and IRDye 680RD Donkey anti-Mouse (LI-COR Biosciences) on an Odyssey imager (LI-COR). NS3-FLAG was normalised to DMSO control and a non-linear regression fitted with Prism 6 (GraphPad) to determine 50% effective concentration (EC_50_).

### 2.4 Cell based screening of small molecules

Selection and maintenance of Huh7.5 cells stably harbouring SGR-feo-NS3-5B (Wyles et al., 2009, Wyles et al., 2007) or SGR-feo-NS2-5B derived from JFH-1 (genotype 2a) and Con1 (genotype 1b) has previously been reported (Shaw et al., 2015). Cells were maintained in Dulbecco's modified Eagle's medium (DMEM) supplemented with 10% FBS, 100 IU penicillin ml^−1^, 100 μg streptomycin ml^−1^, 1% non-essential amino acids and 300 μg/ml G418 in a humidified incubator at 37 °C in 5% CO_2_. For screening, 2 × 10^4^ cells/well were seeded in a 96-well plate in the absence of G418 and compounds were added to 10 μM final concentration by dilution from DMSO stocks. At 72 h, cells were washed in PBS and lysed in 30 μl/well Passive Lysis Buffer (PLB: Promega). Luciferase activity was quantified by automated addition of 40 μl LarI reagent (Promega) and light emission recorded over 6 s using a BMG Labtech Fluostar plate reader. Statistical significance was determined using the Student's *t* test.

### 2.5 Determination of effective concentrations against SGR

For transient SGR experiments, *in vitro* transcripts (2 μg) of firefly luciferase-containing SGR were electroporated into 4 × 10^6^ Huh7.5 cells (Blight et al., 2002) or Huh7 cells at 950 μF and 270 V. 2 × 10^4^ cells/well were seeded in a 96 well plate. At 4 h post electroporation (h.p.e.) media was replaced with media containing compounds.

For cytotoxicity assay, media was removed and cells were incubated in 1 mM thiazolyl blue tetrazolium bromide (MTT) for >2 h. MTT crystals were resuspended in 100 μl DMSO and absorbance at 570 nm quantified using an infinite F50 plate reader (Tecan). Alternatively, cytotoxicity was analysed using the ATPLite kit following the manufacturer's instructions, with light emission quantified using a BMG Labtech Fluostar plate reader.

Firefly luciferase was measured as in Section 2.4. Data was normalised to DMSO controls and EC_50_/CC_50_ determined using Prism 6 (GraphPad).

### 2.6 Western blot analysis of cellular lysates

2 × 10^6^ cells were seeded in a 10 cm dish and incubated as indicated. Cells were washed in PBS and lysed in 100 μl PLB. Clarified lysates were analysed by 15% SDS-PAGE and western blot. Anti-GAPDH (Abcam) (1:20,000) or anti-NS5A (Macdonald et al., 2003) (1:5000) were followed by IRDye 680RD Donkey anti-Mouse or IRDye 800CW Donkey anti-Rabbit (LI-COR BioSciences) (1:10,000) respectively. Imaging was performed using an Odyssey Imager (LI-COR).

### 2.7 Determination of effective concentrations against HCVcc

For HCVcc experiments, 5 μg of Jc1-NLuc (Amako et al., 2015) transcript was electroporated into Huh7.5 cells and treated with compound as described in Section 2.5. Cytotoxicity or NanoLuc (NLuc) activity was measured at 48 h incubation with compound. Cells were lysed as in Section 2.4. Following the addition of 50 μl/well Nano-Glo Luciferase Assay Substrate (Promega) light emission was recorded using a BMG Labtech Fluostar plate reader and data analysed as in Section 2.5.

## 3 Results

### 3.1 Identification of a lead-like small molecule inhibitor of the NS2 autoprotease

**Supplementary Fig. 1:**
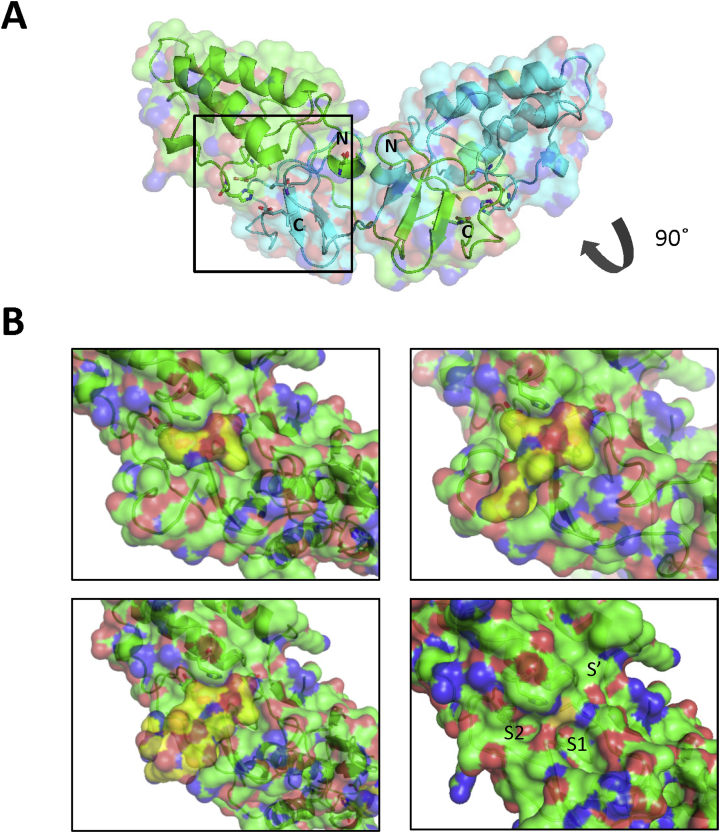
**vHTS applied to a model of the NS2 autoprotease active site.** Structural coordinates of the post-cleaved NS2 C-terminal domain were obtained from the protein databank (PDB 2HD0). A) The active site (boxed) is present at a dimer interface, with one monomer represented with green carbons and the second monomer with light blue carbons. Nitrogen atoms are shown in dark blue and oxygen atoms in red. N- and C-termini are labelled. B) C-terminal residues were sequentially removed (yellow carbons) to access the active site and produce the models NS2Δ_P1−P2_ (top left), NS2Δ_P1−P5_ (top right) and NS2Δ_P1−P10_ (bottom left). S’, S1 and S2 refer to the binding cavities within the active site for the substrate peptide residues P’, P1 and P2, vHTS targeted S1 and S2 regions of the active site (bottom right).

To explore the viability of NS2 autoprotease inhibitors as a novel class of anti-virals, we exploited the availability of a high (2.3 Å) resolution structure representing the post-cleavage NS2 autoprotease (Lorenz et al., 2006). A library of lead-like small molecules was enriched using structure-guided virtual high-throughput screening (vHTS) of >5 × 10^5^ commercially available compounds through a combination of eHITS (Zsoldos et al., 2006) and SPROUT (Gillet et al., 1994) software packages. Structure-guided vHTS was targeted to the NS2 autoprotease structure wherein C-terminal residues were sequentially removed to expose the active site cavity (Supplementary Fig. 1). High scoring compounds with attractive lead-like properties were then screened for activity against NS2-mediated proteolysis in two independent assays.

Firstly, compounds were tested for the ability to block production of a substrate product of the NS2 autoprotease in an *in vitro* assay. This assay, using a recombinant NS2-NS3-FLAG precursor purified under denaturing conditions, measured NS2 autoprotease activity using quantitative western blot to monitor a proteolysis product (NS3-FLAG) (Foster et al., 2010, Shaw et al., 2015, Tedbury and Harris, 2007).

Additionally, compounds were tested for the ability to specifically perturb NS2-dependent HCV genome replication in a cell-based assay. Compounds were tested against cells stably harbouring a HCV sub-genomic replicon (SGR) comprising either NS2-5B or NS3-5B with a firefly luciferase-neomycin phosphotransferase reporter (Shaw et al., 2015, Wyles et al., 2009, Wyles et al., 2007). In this system, SGR-feo-NS2-5B encodes NS3 as part of an NS2-NS3 precursor within the translated polyprotein, hence genome replication is dependent on the NS2 autoprotease activity. In contrast, SGR-feo-NS3-5B lacks the NS2 coding sequence and is thus independent of NS2 autoprotease activity.

**Fig. 1:**
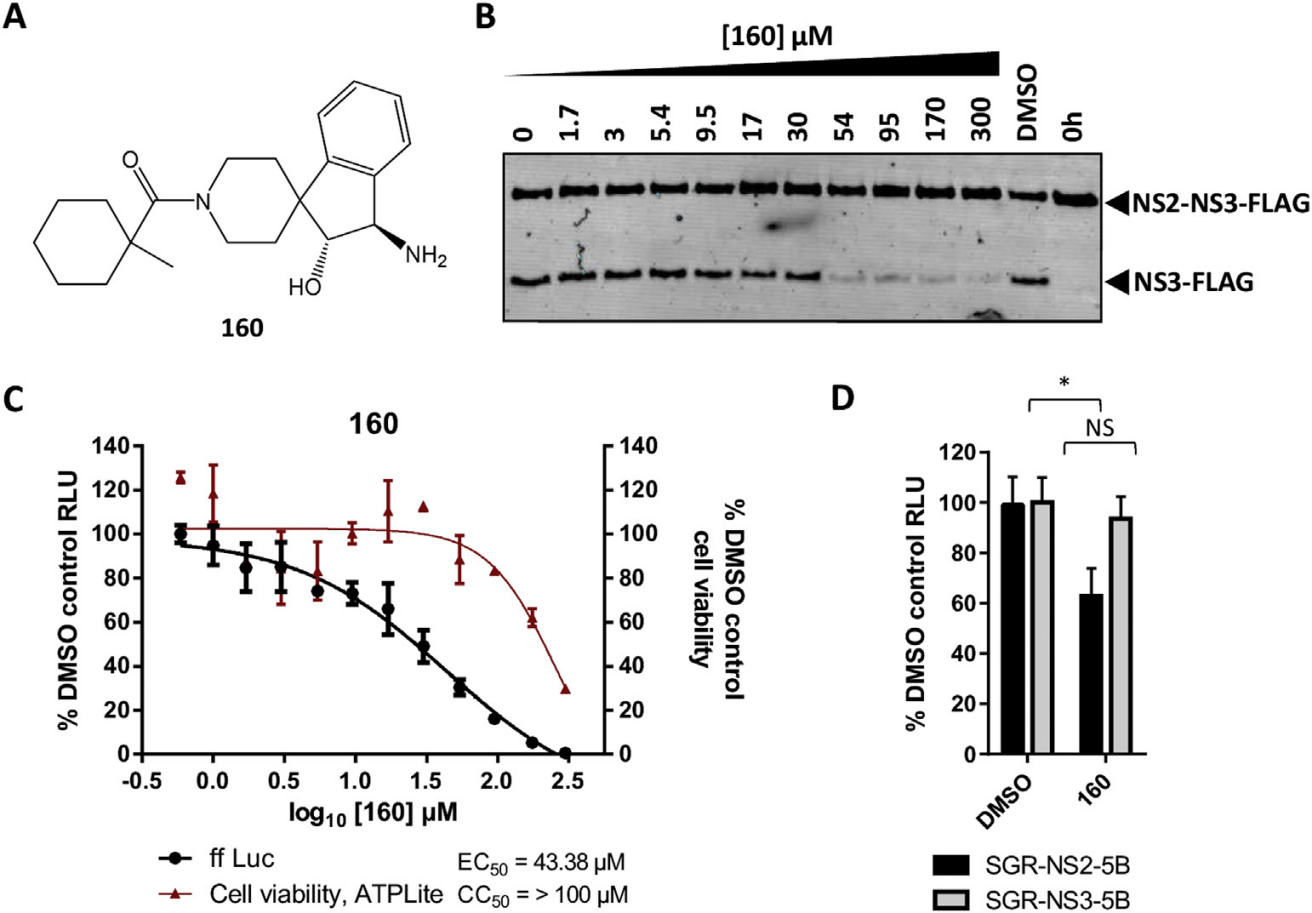
Identification of a lead-like inhibitor of the NS2 autoprotease. A) Chemical structure of active compound **160**. B) Purified recombinant NS2-NS3-FLAG fusion protein was incubated in refolding buffer (Shaw et al., 2015) in the presence of the indicated concentrations of **160**. The presence of the precursor and the NS2 proteolytic product NS3-FLAG was assayed by western blot with an anti-FLAG antibody. C) SGR-feo-NS2-5B(JFH-1) harbouring Huh7.5 cells were treated with the indicated concentrations of **160** for 48 h prior to determination of luciferase activity. Cell viability was measured simultaneously by ATPLite assay. Data represent the mean ± SD of one representative experiment performed in duplicate. D) Addition of **160** (10 μM) reduces luciferase activity in SGR-feo-NS2-5B(Con1) harbouring Huh7.5 cells relative to DMSO control without affecting luciferase activity in SGR-feo-NS3-5B(Con1) harbouring cells. Data show the mean ± SD of two experiments performed in triplicate (RLU – Relative Luciferase Units). Statistical significance was determined by the Student's *t* test (*p = 0.0006).

The compound (2R, 3R)-3-amino-1′-[(1-methylcyclohexyl)carbonyl]-2,3,dihydrospiro[indene-1,4′-piperidine]-2-ol (termed **160** henceforth) (Fig. 1A) was identified as a ‘hit’ in both of these assays. Addition of **160** to the *in vitro* NS2 autoprotease assay reduced production of the NS3-FLAG product in a dose-responsive manner with an EC_50_ of 39 μM (Fig. 1B). In the cell-based assay, **160** also inhibited luciferase activity in cells stably harbouring SGR-feo-NS2-5B(JFH-1) with an EC_50_ of 43.4 μM (Fig. 1C). To confirm that the activity of **160** was not restricted to the genotype 2a JFH-1 isolate, we showed that luciferase activity in Huh7.5 cells stably harbouring the genotype 1b derived SGR-feo-NS2-5B(Con1) was significantly reduced by **160** (10 μM) relative to DMSO control, with no effect on luciferase activity from SGR-feo-NS3-5B(Con1) (Fig. 1D). Importantly therefore, **160** only reduced reporter activity when genome replication was dependent on NS2-mediated processing at the NS2-NS3 junction of the polyprotein.

**Supplementary Fig. 2:**
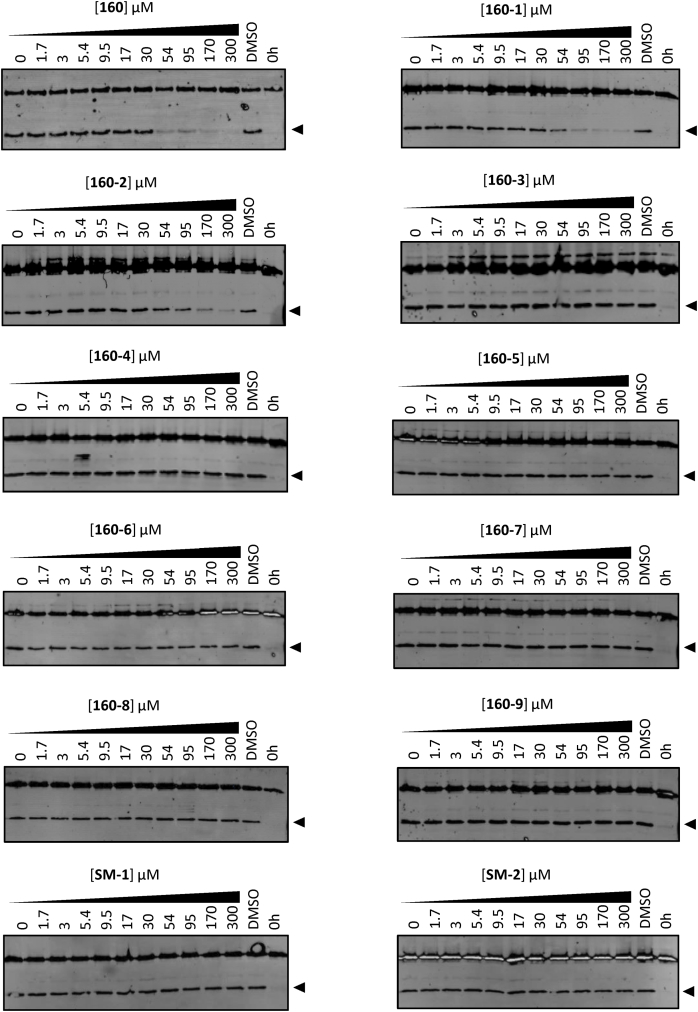
**Structure activity relationship analysis of 160 analogues *in vitro*.** JFH1 NS2-3 autoproteolysis reactions were treated with indicated concentrations of each analogue for 16 h before SDS-PAGE/ Western blot analysis. NS3-FLAG proteolysis product (arrowhead) was quantified to determine EC_50_ (reported in Table 1).

To explore the requirements for activity against the NS2 autoprotease *in vitro*, a range of structurally similar compounds were tested, sourced from both in-house synthesis of variants, and via purchase of commercially available compounds judged to be potentially similar to compound **160** using the ROCS software. Molecules **160–1** and **160–2** were synthesised in-house (for details see supplementary material), while **160–3** to **160–9**, **SM-1** and **SM-2** were commercially available. Compounds were tested for activity against the NS2 autoprotease *in vitro* (Supplementary Fig. 2). Active compounds had no effect on production of the NS3-FLAG proteolysis product when added after the proteolysis reaction was complete (data not shown), confirming that they acted to inhibit proteolysis rather than affecting NS3-FLAG detection by western blot.

As summarised in Table 1, we were able to establish some preliminary structure–activity relationship (SAR) analysis from the small library of molecules that were screened. Thus, activity of **160** against the NS2 autoprotease *in vitro* did not require the presence of the primary alcohol and amine groups at the stereocentres of the indene core in **160**. However, activity was found to be crucially dependent on the presence of the cyclohexane portion in **160** and is enhanced by the presence of a 1-(methylcyclohexyl) moiety as in **160** and **160–1.** This would indicate that, whilst hydrophobic interactions between this portion of the inhibitor and protein would appear to be important, there is also a requirement for shape complementarity. These observations begin to map the features of **160** which contribute to activity against the NS2 autoprotease.

### 3.2 Activity of 160 against a transient NS2-5B SGR

**Fig. 2:**
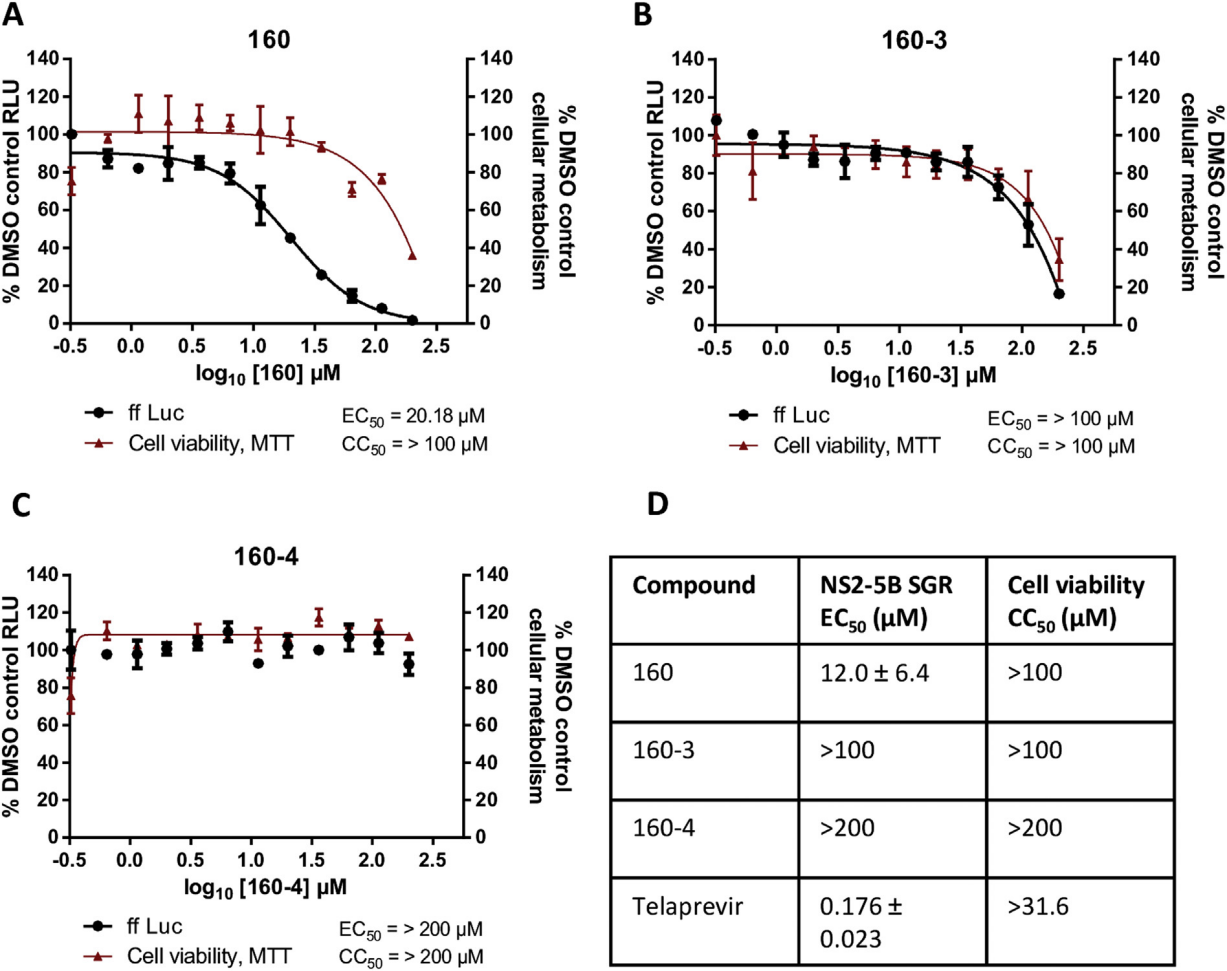
Activity of 160 and analogues against NS2-dependent genome replication. SGR-luc-NS2-5B(JFH-1) RNA was electroporated into Huh7.5 cells which were treated with indicated concentrations of **160** (A) or the inactive derivatives **160–3** (B) or **160–4** (C) at 4 h.p.e. At 48 h.p.e cell viability was quantified by MTT assay and luciferase activity was measured and plotted relative to DMSO control. Data represent the mean ± SD of one representative experiment performed in duplicate. (D) EC_50_ and CC_50_ for indicated compounds determined across several experimental repeats. Additional primary data is presented in Supplementary Fig. 3. Mean ± SD are reported.

Inhibition of both NS2-mediated proteolysis *in vitro* (Fig. 1B), and NS2-dependent HCV genome replication in a cell based assay (Fig. 1C) by **160** exhibited similar EC50 values (39/43.4 μM respectively). We considered that the relatively low efficacy of **160** in the cell-based assay might be due to the fact that these cells had been selected to harbour a stable SGR and thus they contained pre-existing, replication complexes whose activity would not be dependent on NS2-3 cleavage. To test this we performed a transient replication assay in which *in vitro* transcripts of a JFH-1 derived luciferase-containing SGR (SGR-luc-NS2-5B(JFH-1)) (Tedbury et al., 2011) were electroporated into the HCV permissive Huh7.5 cell line (Blight et al., 2002) and treated for 48 h with a range of concentrations of **160**. Cytotoxicity was quantified by MTT assay and replication assayed by measuring luciferase activity. A representative assay is presented (Fig. 2A), demonstrating that treatment with **160** produced a dose responsive reduction in luciferase activity. Over four biological replicates (Fig. 2D) **160** did indeed exhibit an improved EC_50_ of 12.0 ± 6.4 μM, compared to the activity observed in the stable SGR-harbouring cells. At higher concentrations of **160**, some cytotoxic effects were observed (Fig. 2A, D). However, due to the limitation in the achievable concentration of **160** and derivatives (300 μM), it was not possible to calculate an accurate CC_50_ value, although the data indicate a selectivity index of >5.

In contrast, **160–3** and **160–4**, derivatives inactive against the NS2 autoprotease *in vitro* (Supplementary Fig. 2), did not exhibit any specific effect against replication. For **160**–**3** luciferase activity closely correlated with the degree of cytotoxicity indicating a lack of any specific effect on SGR genome replication (Fig. 2B, D). In contrast **160**–**4** had no cytotoxicity or effect on luciferase activity up to 200 μM (Fig. 2C, D).

**Supplementary Fig. 3:**
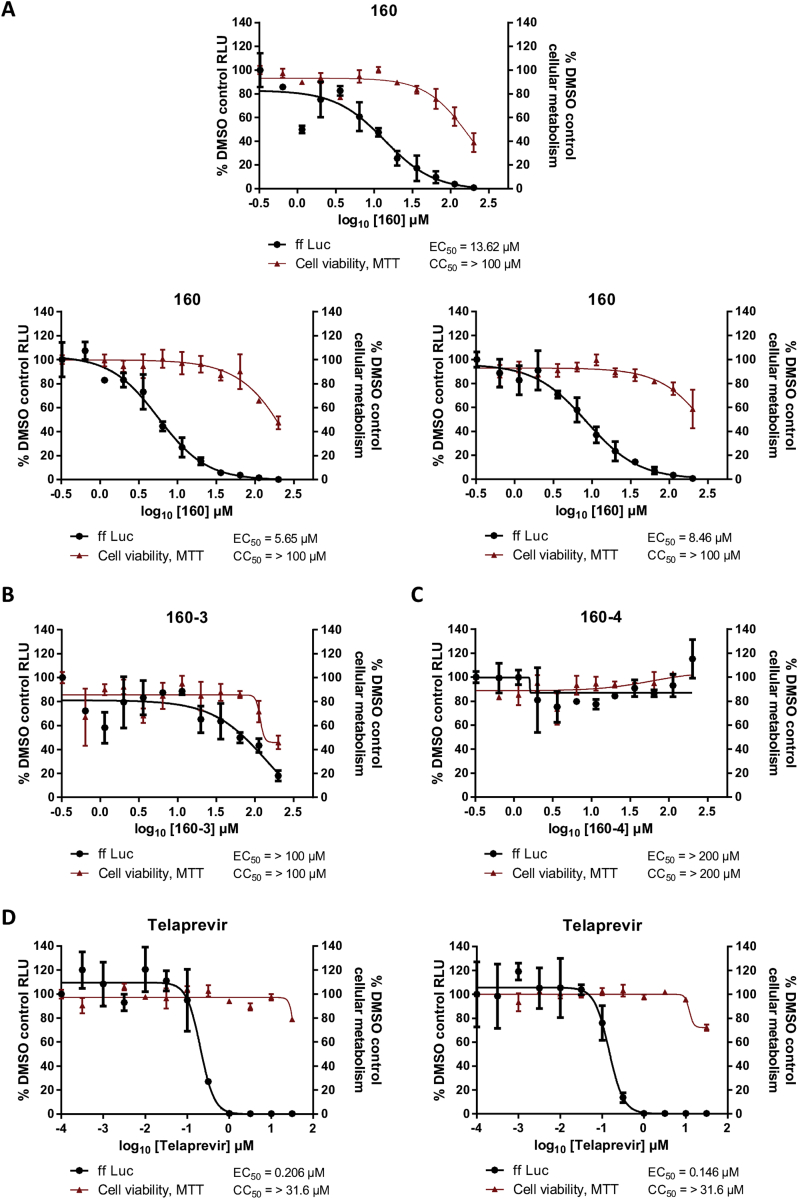
**Activity of 160 and analogues against NS2-dependent genome replication.** SGR-luc-NS2-5B(JFH-1) RNA was electroporated into Huh7.5 cells which were treated with indicated concentrations of **160** (A) or the inactive derivatives **160-3** (B) or **160-4** (C) at 4 h.p.e. At 48 h.p.e cell viability was quantified by MTT assay and luciferase activity was measured and plotted relative to DMSO control. Data represent the mean ± SD of individual experiments performed in duplicate. (D) As a control the NS3 protease inhibitor Telaprevir was used in the same assay.

**Fig. 3:**
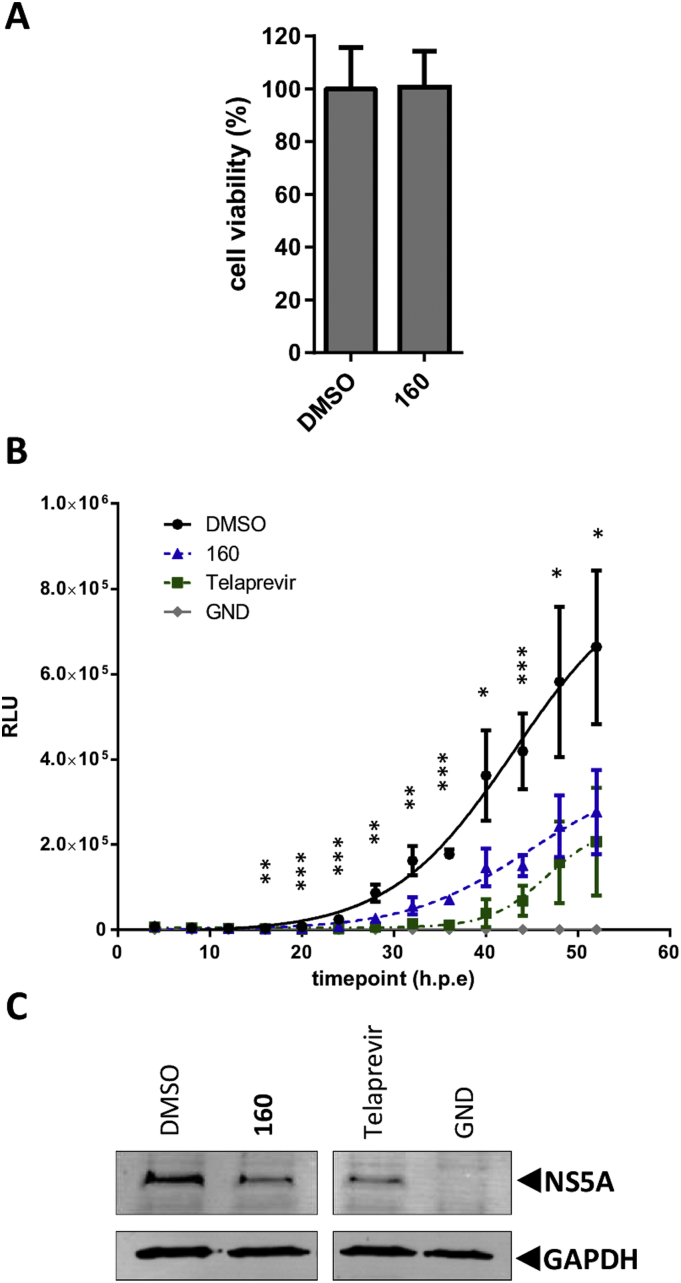
160 inhibits replication of a NS2-5B sub-genomic replicon in the absence of cytotoxicity. A) Huh7.5 cells treated with DMSO control or an EC_50_ concentration of **160** (25 μM) for 48 h before quantifying viable cells using the ATPLite assay. Data is normalised to DMSO control and represents the mean ± SD of three experimental repeats performed in triplicate. B) SGR-luc-NS2-5B(JFH-1) or a polymerase defective control (GND) were electroporated into Huh7.5 cells prior to treatment with DMSO control, **160** (25 μM) or Telaprevir (100 nM) and assay for luciferase activity at the indicated time points. Data represent the mean ± SD of two experimental repeats performed in triplicate. Statistical significance was determined by the Student's *t* test (***p ≤ 0.0001, **p ≤ 0.0002, *p ≤ 0.0023). C) Cell lysates from B at 52 h.p.e. were analysed for NS5A and GAPDH by western blot.

To confirm an inhibitory effect of **160** against NS2-dependent genome replication in the absence of cytotoxic effects, SGR-luc-NS2-5B(JFH-1) electroporated cells were treated with a concentration of **160** (25 μM) that had no observed cytotoxicity as measured either by MTT (Fig. 2A, Supplementary Fig. 3), or ATPlite assay (Fig. 3A). **160** was added 4 h.p.e and luciferase activity was monitored every 4 h for a further 48 h. Addition of **160** led to a reduction in luciferase activity and a lag in detectable luciferase activity relative to DMSO control (Fig. 3B). Addition of an intermediate concentration (100 nM) of Telaprevir produced a comparable profile.

Cell lysates at 52 h.p.e were analysed by western blot for NS5A and GAPDH. Both **160** and Telaprevir caused a comparable reduction in NS5A relative to DMSO control (Fig. 3C). These results demonstrate that **160** inhibits NS2-dependent HCV genome replication in the absence of cytotoxicity.

### 3.3 160 exhibited anti-viral activity

**Fig. 4:**
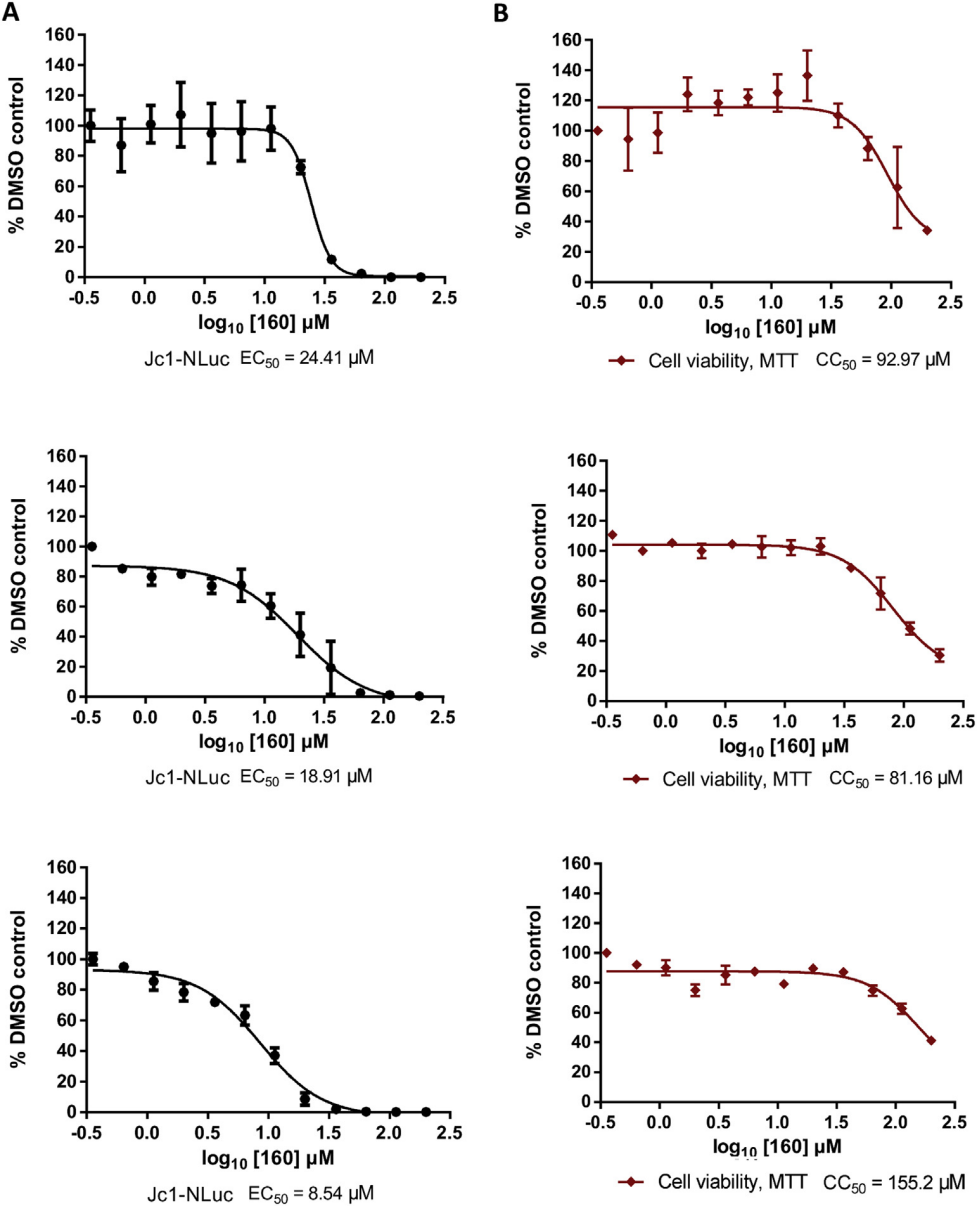
Anti-viral activity of 160. Jc1-NLuc RNA was electroporated into Huh7.5 cells and treated with a range of concentrations of **160**. A) 48 h.p.e. NLuc activity was quantified to assess anti-viral effects. B) 48 h.p.e cellular metabolism was quantified by MTT assay (Cell viability, MTT). Data is normalised to DMSO control with the mean ± SD of three experiments performed in duplicate shown. Mean EC_50_ = 17.3 ± 8.1 μM. Mean CC_50_ = 109.8 ± 40.0 μM.

We next assessed the antiviral activity of **160** in an infectious system, using a derivative of the genotype 2a chimeric Jc1 construct that encodes a Nano-luciferase reporter (Jc1-NLuc) (Amako et al., 2015). Jc1-NLuc RNA was electroporated into Huh7.5 cells and treated with **160** from 4 to 48 h.p.e. Three biological replicates of this experiment are shown in Fig. 4 and demonstrate that **160** treatment resulted in a dose-dependent reduction in NLuc reporter activity with an EC_50_ of 17.3 ± 8.1 μM that was comparable to that observed against SGR (Fig. 4A). Cytotoxicity of **160** was simultaneously assessed by MTT assay (Fig. 4B) and was comparable to that observed in SGR (Fig. 2). These findings demonstrate that the small molecule **160**, which blocks NS2-NS3 processing *in vitro*, can function as an anti-viral compound.

## 4 Discussion

Recent advances in all-oral, interferon-free HCV therapeutic regimes have involved the use of DAAs targeting HCV-encoded enzyme activities; namely inhibitors targeting NS3/4A protease and NS5B RNA polymerase. Small molecule inhibitors of the NS3 helicase have also been reported but remain in pre-clinical stages. Thus the only HCV-encoded enzyme activity which has not been targeted with DAAs is the protease activity encoded within the C-terminus of NS2.

We report the identification of a lead-like small compound, **160**, which blocks NS2-mediated proteolysis at the NS2-NS3 junction *in vitro*. SAR analysis has begun to inform on the active pharmacophore of this series: **160**, and its active analogues **160–1** and **160–2**, represent an interesting chemotype that could be expanded to explore additional, more potent, inhibitors of NS2-mediated proteolysis. Treatment with **160** reduced reporter activity in a SGR system where genome replication was dependent on NS2 autoprotease activity (SGR-NS2-5B) without affecting reporter activity in a SGR system that lacks NS2 (SGR-NS3-5B) (Fig. 1B). The EC_50_ of **160** against SGR-NS2-5B was 12.0 ± 6.4 μM (Fig. 2D), an improved potency compared to previously reported NS2 inhibitors (Shaw et al., 2015). Importantly, **160** also inhibited viral replication in the context of infectious virus, reducing reporter activity at concentrations where cellular metabolism remains unaffected (Fig. 4).

The activity of **160** had a narrow selectivity index (>5), as modest cytotoxic effects were observed at ≥ 95 μM and at the maximal attainable concentration (300 μM), cell viability was reduced to 40%. However, at an intermediate concentration of **160** (25 μM) both reporter activity and NS5A expression were reduced in the absence of detectable cytotoxicity (Fig. 3). Furthermore, SAR demonstrated that the derivatives **160–3** and **160–4** were inactive in both the *in vitro* NS2 autoprotease and cell-based NS2-dependent replication assays. **160–3** only showed reduced reporter activity at ≥ 95 μM which correlated with decreased cellular metabolism, hence the compound possessed no selective anti-viral activity. This suggests that a hydrophobic group in place of the 1-methyl cyclohexane portion of **160** was associated with cytotoxicity. However, while both an isopropyl or 1-methyl substituted cyclohexane at this position are responsible for cytotoxic effects, only a 1-methyl cyclohexane moiety imparts activity against NS2-mediated proteolysis *in vitro* and against NS2-dependent HCV genome replication. Further modifications to this region may help to increase the selectivity index of future compounds.

In conclusion, our study has identified a small molecule (**160**) that selectively inhibits the cleavage of the NS2-3 precursor. Although the potency of **160** is not sufficient to impact on current therapeutic regimes, it provides a starting point for further development, and importantly also represents proof of principle that this proteolytic cleavage is a valid and tractable target for future antiviral strategies to enhance the breadth of HCV therapies.

## Figures and Tables

**Table 1:** Structure activity relationship analysis of 160. EC_50_ calculated from 10-point dose response treatment of JFH-1 NS2-3 *in vitro* proteolysis reactions (Supplementary Fig. 2).* Mean ± SD of three experimental repeats.

Compound	Structure	JFH1 NS2-3 EC_50_ (μM)
160		44.6 ± 6.2 *
160–1		43.0 ± 0.5 *
160–2		68.4 ± 5.6 *
160–3		>300
160–4		>300
160–5		>300
160–6		>300
160–7		>300
160–8		>300
160–9		>300
SM-1		>300
SM-2		>300
