# Supplementary material for: Identification of a lead like inhibitor of the hepatitis C virus non-structural NS2 autoprotease
Source: Antiviral Res. 2015 Dec;124:54–60. doi: 10.1016/j.antiviral.2015.10.001 (PMC4678293; doi:10.1016/j.antiviral.2015.10.001)
Supplement: Supplementary file 1 [file mmc1.docx]

**Identification of a lead like inhibitor of the hepatitis C virus non-structural 2 autoprotease**

Joseph Shaw^a,b^, Mark Harris^b^, Colin W.G. Fishwick^a¥^

^a^ School of Chemistry, University of Leeds, Leeds, United Kingdom, LS2 9JT.

^b^ School of Molecular and Cellular Biology, Faculty of Biological Sciences, University of Leeds, Leeds, United Kingdom, LS2 9JT.

^¥^ Corresponding author

Postal address: School of Chemistry, University of Leeds, Leeds, United Kingdom, LS2 9JT

Email: [C.W.Fishwick@leeds.ac.uk](mailto:C.W.Fishwick@leeds.ac.uk)

Tel: +44 113 343 6510

**Supplementary Material**

**Synthetic procedures**

**1-[(1-Methylcyclohexyl)carbonyl]-spiro[indene-1,4’-piperidine] (160-1)**

1-Hydroxybenzotriazole (457.1 mg, 3.383 mmol) and 1-(3-dimethylaminopropyl)-3-ethylcarbodiimide (648.5 mg, 3.383 mmol) were added to a stirred solution of 1-methyl-1-cyclohexanecarboxylic acid (352.8 mg, 2.481 mmol) in DMF (12.5 ml), and the resulting solution was stirred at room temperature for 1 hour. 4-Spiroindene-piperidine hydrochloride (0.5 g, 2.255 mmol) and TEA (376 µl, 2.706 mmol) were then added and stirred at room temperature for 16 hours. Following evaporation of the solvent the resulting residue was partitioned between EtOAc (20 ml) and water (2 x 20 ml) followed by brine (2 x 20ml) and the organic extracts were dried (MgSO_4_) before the solvents were removed under reduced pressure. The resulting solid was purified via column chromatography to yield the title compound (294 mg, 0.950 mmol, 42 %) as a colourless powder; m.p 116-118 °C ; R_f_ 0.88 (10 % MeOH/DCM); δ_H_ (500 MHz, CDCl_3_); 7.30 (m, 4H), 6.86 (dd, (*J* = 24.1, 5.7, 2H), 4.54 (d, *J* = 14.3, 2H), 3.20 (d, *J* = 13.0, 2H), 2.20-1.89 (m, 4H), 1.59 (m, 5H), 1.52-1.12 (m, 10H); *m/z* (ES); (found MH^+^ 310.224; C_21_H_27_NO requires *MH* 310.21); LC-MS (RT= 2.15 min, *m/z* found MH^+^ 310.2).

**1’-[(Cyclohexyl)carbonyl]-spiro[indene-1,4’-piperidine] (160-2)**

1-Hydroxybenzotriazole (182.8 mg, 1.353 mmol) and 1-(3-dimethylaminopropyl)-3-ethylcarbodiimide (259.4 mg, 1.353 mmol) were added to a stirred solution of cyclohexane carboxylic acid (123 µl, 0.992 mmol) in DMF (5 ml). The solution was stirred at room temperature for 15 minutes. 4-Spiroindene-piperidine hydrochloride (200 mg, 0.902 mmol) and TEA (150 µl, 1.082 mmol) were then added and the resulting solution was stirred at room temperature for 16 hours. Following evaporation of the solvent the resulting residue was partitioned between EtOAc (20 ml) and water (2 x 20 ml) followed by brine (2 x 20ml) and the organic extracts were dried (MgSO_4_) before the solvents were removed under reduced pressure. The resulting solid was purified via column chromatography to yield the title compound (148.8 mg, 0.504 mmol, 56 %) as a colourless powder; m.p 134-135 °C ; R_f_ 0.80 (10 % MeOH/DCM); δ_H_ (500 MHz, CDCl_3_); 7.36-7.18 (m, 4H), 6.84 (dd, (J = 27.0, 5.7 , 2H), 4.70 (d, *J* = 13.7, 1H), 4.04 (d, *J* = 13.3, 1H), 3.40 (t, *J* = 12.3, 1H), 3.00 (t, *J* = 12.2, 1H), 2.56 (t, *J* = 11.5, 1H), 2.05-1.92 (m, 2H), 1.81 (m, 4H), 1.72 (s, 1H), 1.68-1.36 (m, 5H), 1.29 (m, 3H); *m/z* (ES); (found MH^+^ 296.192; C_20_H_25_NO requires *MH* 296.2); LC-MS (RT= 2.20 min, *m/z* found MH^+^ 296.2).

**Supplementary Figure legends**

**Supplementary Figure 1: vHTS applied to a model of the NS2 autoprotease active site.** Structural coordinates of the post-cleaved NS2 C-terminal domain were obtained from the protein databank (PDB 2HD0). A) The active site (boxed) is present at a dimer interface, with one monomer represented with green carbons and the second monomer with light blue carbons. Nitrogen atoms are shown in dark blue and oxygen atoms in red. N- and C-termini are labelled. B) C-terminal residues were sequentially removed (yellow carbons) to access the active site and produce the models NS2Δ_P1-P2_ (top left), NS2Δ_P1-P5_ (top right) and NS2Δ_P1-P10_ (bottom left). S’, S1 and S2 refer to the binding cavities within the active site for the substrate peptide residues P’, P1 and P2, vHTS targeted S1 and S2 regions of the active site (bottom right).

**Supplementary Figure 2: Structure activity relationship analysis of 160 analogues *in vitro*.** JFH1 NS2-3 autoproteolysis reactions were treated with indicated concentrations of each analogue for 16 h before SDS-PAGE/ Western blot analysis. NS3-FLAG proteolysis product (arrowhead) was quantified to determine EC_50_ (reported in Table 1).

**Supplementary Figure 3. Activity of 160 and analogues against NS2-dependent genome replication.** SGR-luc-NS2-5B(JFH-1) RNA was electroporated into Huh7.5 cells which were treated with indicated concentrations of **160** (A) or the inactive derivatives **160-3** (B) or **160-4** (C) at 4 h.p.e. At 48 h.p.e cell viability was quantified by MTT assay and luciferase activity was measured and plotted relative to DMSO control. Data represent the mean ± SD of individual experiments performed in duplicate. (D) As a control the NS3 protease inhibitor Telaprevir was used in the same assay.
